# A Time-Dependent Analysis of Association between Acupuncture Utilization and the Prognosis of Ischemic Stroke

**DOI:** 10.3390/healthcare10050756

**Published:** 2022-04-19

**Authors:** Sung-Ryul Choi, Eun-San Kim, Bo-Hyoung Jang, Boyoung Jung, In-Hyuk Ha

**Affiliations:** 1 Jaseng Hospital of Korean Medicine, Seoul 06110, Korea; csrs0102@naver.com; 2 Jaseng Spine and Joint Research Institute, Jaseng Medical Foundation, Seoul 06110, Korea; eunsan.research@gmail.com; 3 Department of Preventive Medicine, College of Korean Medicine, Kyung Hee University, Seoul 02447, Korea; bhjang@khu.ac.kr; 4 Department of Health Administration, Hanyang Women’s University, Seoul 04763, Korea; yqh630@hywoman.ac.kr

**Keywords:** acupuncture, ischemic stroke, mortality, complications, time-dependent analysis

## Abstract

This study investigated the time-dependent characteristics of acupuncture and analyzed the association between acupuncture utilization and mortality rates, readmission rates, and complications among ischemic stroke patients. Data from the National Health Insurance Service-National Sample Cohort 2.0 from South Korea were used to track patients with subacute and chronic ischemic stroke, who had survived more than one month after onset, between 2010 and 2013. A total of 2299 patients were followed up until 2015. At baseline, the acupuncture group (*n* = 195) and the control group (*n* = 2104) had similar ages (acupuncture group: 69.0 ± 11.1 years; control group: 68.5 ± 11.8 years), but the acupuncture group had more comorbidities (Charlson comorbidity index; acupuncture group: 4.7 ± 2.1, control group: 4.3 ± 2.4). According to time-dependent Cox regression survival analysis, acupuncture treatment was associated with low hazard ratios (HR) for death (HR: 0.32; 95% confidence interval (CI): 0.18–0.60), fewer composite complications (HR: 0.34; 95% CI: 0.21–0.53), and reduced urinary tract infection (HR: 0.24; 95% CI: 0.11–0.54). Many acupuncture session sensitivity analyses were performed to assess the robustness using different criteria to define the acupuncture group, and the results were consistent with those of the main analysis. Therefore, acupuncture treatment might be associated with lower mortality rates and the prevention of complications after ischemic stroke.

## 1. Introduction

Stroke is associated with a high mortality rate worldwide and is a major cause of acquired disability [1]. In South Korea, stroke is ranked as the fourth major cause of death [2]. After a stroke event, 18.6% of patients died within one year [3], which is extremely higher than the total mortality rate among Koreans (593.9 per 100,000 people) [2]. Readmission attributable to recurrence is common among ischemic stroke survivors, with an estimated 31% of patients being readmitted within 1 year after the initial stroke [4]. Complications are important factors for the prognosis of stroke patients. For example, pneumonia is a common complication among stroke patients [5]. In addition, the incidence rate of hip fracture among female stroke patients aged ≥50 years is 1495.2 per 100,000 patients [6], which is about 10 times higher than the incidence rate among all women aged ≥50 years (146.38 per 100,000 women) [7]. Furthermore, urinary tract infection [8] and decubitus ulcer [9] are also key complications that need to be managed. Those complications may prolong the hospital stay, increase medical costs, exacerbate post-discharge disability, and increase mortality rates [10].

Acupuncture has been used to rehabilitate patients after stroke in East Asia. Acupuncture can be an effective, safe intervention for stroke rehabilitation, easily combined with conventional treatment. In 2016, Cochrane reviewed 31 trials with a total of 2257 sub-acute or chronic stroke patients and concluded that acupuncture may have a beneficial effect on activity of daily living, neurological impairments, cognitive function, depression, swallowing function, and pain [11]. For example, when comparing combination of conventional treatment and acupuncture with conventional treatment alone, dependency significantly improved (measured by Barthel index: 9.19; 95% confidence interval (CI): 4.34 to 14.05) at the end of the treatment period (one to 24 weeks) and this effect persists at the end of the follow-up (3 months) [11]. In addition, Wang, et al. [12] studied the effectiveness of acupuncture on motor function with 134 subacute (i.e., 30–90 days after onset) stroke patients. They found that neurological motor function significantly improved with acupuncture, especially on lower-limb function (measured by Fugl–Meyer Assessment: 5.72; 95% CI: 2.74–8.69). Both studies reported little incidence of adverse events [11,12]. Chavez et al. [13] reviewed 40 studies to explain the mechanisms of beneficial effects of acupuncture on stroke. They concluded that acupuncture might be associated with promotion of neurogenesis and cell proliferation in the central nervous system, regulation of cerebral blood flow in the ischemic area, anti-apoptosis in the ischemic area, regulation of neurochemicals, and improvement of impaired long-term potentiation and memory after stroke.

Unlike surgery or hospitalization, which are essentially performed once after stroke onset, acupuncture can be performed several times over a long period after stroke onset. Because of its time-dependent characteristics, the outcomes of acupuncture can vary with the patient’s adherence to treatment; For example, Zhang et al. [14] studied the effect of acupuncture on mortality of stroke patients. They concluded that the benefit of acupuncture was noted in the subgroup (odds ratio (OR), 0.68; 95% CI: 0.47–0.98) who complied with protocol (i.e., receiving ≥10 sessions of acupuncture). Moreover, ignoring time-dependent characteristics could lead to biased estimates [15,16], so several articles have suggested using appropriate time-varying analysis models to solve this problem [16,17,18]. Therefore, we aimed to investigate the time-dependent characteristics of acupuncture treatment and analyze the association between acupuncture utilization and the prognosis of ischemic stroke patients using time-dependent analysis.

## 2. Materials and Methods

### 2.1. Data Source

The National Health Insurance Service-National Sample Cohort (NHIS-NSC) 2.0 was established by the NHIS as a population-based cohort for 2002–2015 [19]. NHIS-NSC was established by sampling 2% of the Korean population. NHIS data contain personal information, population statistics, and treatment data based on insurance claims submitted to the NHIS by medical institutions that provide inpatient and outpatient treatments. Such claims data include patient demographics, diagnostic codes, treatment details, test details, prescribed medications, and medical fees. The Korean Classification of Diseases (Seventh Revision) was used to code diagnoses in the NHIS-NSC database, and it is based on the International Classification of Diseases (Tenth Revision, Clinical Modification). Because of ethical considerations, personally identifiable cohort information was encrypted. The study protocol was approved by the Institutional Review Board of Jaseng Hospital of Korean Medicine (JASENG 2019-05-010). The requirement for informed consent was waived by the board. All methods were performed in accordance with relevant guidelines and regulations.

### 2.2. Design and Definition of Acupuncture Utilization

Acupuncture treatment was defined using reimbursement records. The definition of acupuncture treatment for the rehabilitation of ischemic stroke patients is inconsistent. Based on a recent systematic review [11], we only included invasive acupuncture treatment and excluded non-invasive methods such as moxibustion and cupping (detailed codes are described in Table S1).

The current study design is presented in Figure S1, and a detailed example is presented in Table S2. In short, the follow-up time is stratified by 3 months. Later, the compliance to acupuncture treatment is assessed by each stratified period. If a patient’s compliance is higher than specified criterion, the patient is allocated to an acupuncture group during that period. However, clear criteria regarding the number of acupuncture sessions required for ischemic stroke patients remain undetermined. Previous studies have used varying criteria, such as two [20], three [21], six [22], and 20 or more acupuncture sessions [23]. Criteria including a low number of sessions could result in underestimated outcomes of acupuncture treatment. However, applying criteria including 20 sessions within 3 months [23] to real-world data could lead to selection bias. Hence, we defined the acupuncture group as patients who received weekly acupuncture treatment weekly (at least 13 sessions within 3 months), and adopted other criteria (i.e., 3, 6, 10, 18, 20) in the sensitivity analysis. Furthermore, we performed a descriptive analysis of the adherence to acupuncture treatment and cross-over.

Removing the time prior to receiving acupuncture treatment for the acupuncture group from the analysis could cause underestimation of the survival period of the control group, which could lead to an immortal time bias. For robustness, the period prior to acupuncture assignment was analyzed for the control group [24]. The control group was defined as patients who did not receive any acupuncture treatment during each time window. If a patient in the control group received at least one acupuncture session, then data after that point were censored. Patients were recruited from 1 January 2010 to 31 December 2013. The follow-ups ended on 31 December 2015. Because only nationally licensed KM doctors can file NHIS claims for acupuncture treatment reimbursement, the quality of acupuncture treatment was considered to have been indirectly controlled.

### 2.3. Study Population

The study population in this retrospective cohort study comprised patients with subacute and chronic ischemic stroke. Patients diagnosed with ischemic stroke (Korean Classification of Diseases, Seventh Revision; code I63) for the first time between January 2010 and December 2013 (*n* = 10,865) were selected. In the dual medical system in South Korea, physicians who practice KM and those who practice Western medicine diagnose and treat patients separately; furthermore, these physicians used different diagnostic codes before 2010. Therefore, data before 2010 were excluded. Moreover, we determined that the follow-up period required to observe long-term outcomes should be at least two years [21]. Accordingly, patients with ischemic stroke were included up to 2013. Patients were included if they were 40 years or older at the time of stroke onset (*n* = 10,634) and hospitalized for at least 1 day after stroke onset (*n* = 4170).

To validate the diagnosis of stroke, only patients with neuroimaging records, such as computed tomography or magnetic resonance imaging at the time of onset, were selected (*n* = 3716). Among these patients, only those who received antithrombotic drugs (including anticoagulants or antiplatelets; anatomical therapeutic chemical code: B01) a day before or after onset were selected (*n* = 3416).

**Figure 1:**
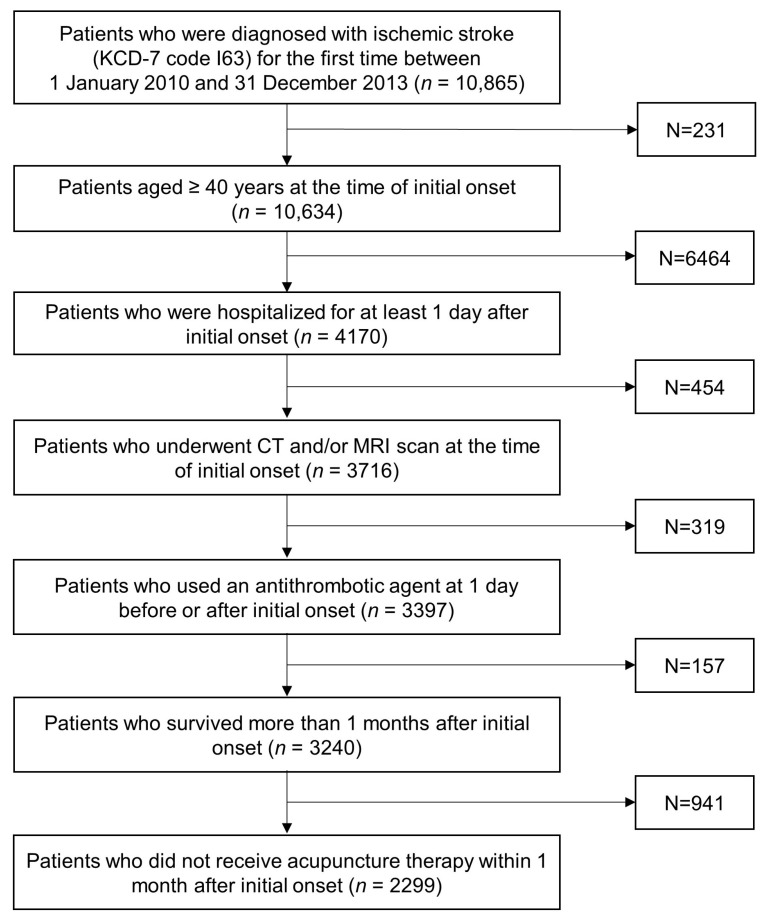
Flowchart of the study population. KCD: Korean Classification of Diseases; CT: computed tomography; MRI: magnetic resonance imaging.

Following the previous literature on stroke rehabilitation, we included patients in subacute and chronic phase [11]. Accordingly, patients who died within one month after stroke onset were excluded (*n* = 3240). In addition, patients who received acupuncture during acute phase were excluded (*n* = 2299) (Figure 1). Follow-up started one month after stroke onset.

### 2.4. Covariates

Data regarding gender, age, income, residence, insurance, and disability were identified at one month after onset, which was the time for cohort entry. Major risk factors (e.g., Charlson comorbidity index, diabetes, hypertension, and hyperlipidemia) were adjusted from 1 year prior to entry into the study until diagnosis. Decubitus ulcer, gastrointestinal bleeding, and femur fracture were not adjusted because of the excessively small sample sizes (all *n* < 3 in the acupuncture group at baseline). Medical interventions that could reflect the severity at onset were also adjusted, including admission to tertiary hospitals, admission day, nasogastric intubation, urinary catheterization, intensive care unit stay, and readmission within one month from stroke onset (detailed codes are described in Table S1).

### 2.5. Outcomes

The assessed outcomes included all-cause deaths, readmissions because of ischemic stroke recurrence, and complications. Readmission was defined as hospitalization for at least 1 day with a primary diagnosis of ischemic stroke and neuroimaging records after the onset of the first ischemic stroke. Regarding complications, pneumonia, urinary tract infection (UTI), decubitus ulcer, gastrointestinal bleeding, and femur fracture were considered for both composite and distinct outcomes (detailed codes are described in Table S1). The incidence per 100,000 person-days of each outcome was calculated. For gastrointestinal bleeding and femur fracture, only the incidence was analyzed because of the small number of events.

### 2.6. Statistical Analysis

Events are presented as cases and incidences. The occurrence of events in the groups was determined according to the groups allocated every 3 months. The incidence was calculated per 100,000 person-days. Because group allocation was time-varying, an extended Kaplan–Meier graph [25] was used to present the cumulative incidence for each group.

The hazard ratio (HR) for an event was estimated by Cox regression. The proportional hazard assumption for all covariates was tested using the Schoenfeld residuals significance (*p* < 0.05) and log–log graphs. If either of these two did not satisfy this assumption, then the interaction term between that covariate and time was additionally adjusted. All analyses were performed using R Studio (version 1.0.136; RStudio, Inc., Boston, MA, USA).

### 2.7. Sensitivity Analysis

Three different sensitivity analyses were performed. We used propensity score matching because of the unmatched numbers in the acupuncture and control groups. The ratio of the acupuncture group and control group was 1:2. Nearest neighbor matching was performed with a caliper width of 0.2. All baseline covariates were included to estimate the propensity score.

Next, we changed the criteria for defining the acupuncture group; the minimum number of acupuncture sessions within 3 months used for the sensitivity analyses were 3 [21], 6 (the median of the cumulative number of acupuncture sessions received during each time window among patients prescribed acupuncture based on a prior study [22]), 10 (half the number of sessions used during a previous randomized controlled study [23]), 18 (the 75th percentile of the cumulative number of acupuncture sessions received by each time window among patients prescribed acupuncture), and 20 [23].

Since there was a possibility that only patients with relatively mild stroke severity would receive acupuncture as the follow-up progressed, the follow-up was changed from a minimum of 2 years to a maximum of 6 years, and changes in the results were evaluated accordingly.

## 3. Results

### 3.1. Baseline Characteristics

The study population comprising patients with subacute and chronic ischemic stroke (*n* = 2299) was divided into the acupuncture group (*n* = 195; 8.5%) and control group (*n* = 2104; 91.5%) at baseline. The proportion of females and age were similar in the acupuncture and control groups (42.1% and 69.0 ± 11.1 years vs. 42.4% and 68.5 ± 11.8 years). Overall, the acupuncture group had higher rates of comorbidities, as indicated by the Charlson comorbidity index, than did the control group (acupuncture: 4.7 ± 2.1; control: 4.3 ± 2.4). Previous medical interventions were also higher in the acupuncture group than in the control group (Table 1).

### 3.2. Acupuncture Treatment

The acupuncture group at baseline comprised 195 patients (8.5%). Among the 195 patients who were assigned to the acupuncture group at baseline, 179 of them (91.8%) subsequently discontinued acupuncture treatment. The median time until discontinuation was 179 days (95% CI: 92–184 days). On the other hand, 152 (6.6%) patients from the control group did cross over to acupuncture group. As a result, 347 patients (15.1%) were allocated to the acupuncture group at least once during follow-up. A total of 347 patients received 32,777 sessions during follow-up (94.5 ± 114 sessions per one patient). Acupuncture was primarily administered for cerebral infarction sequelae and musculoskeletal diseases, such as low back pain (Table S3).

### 3.3. Outcomes

**Figure 2:**
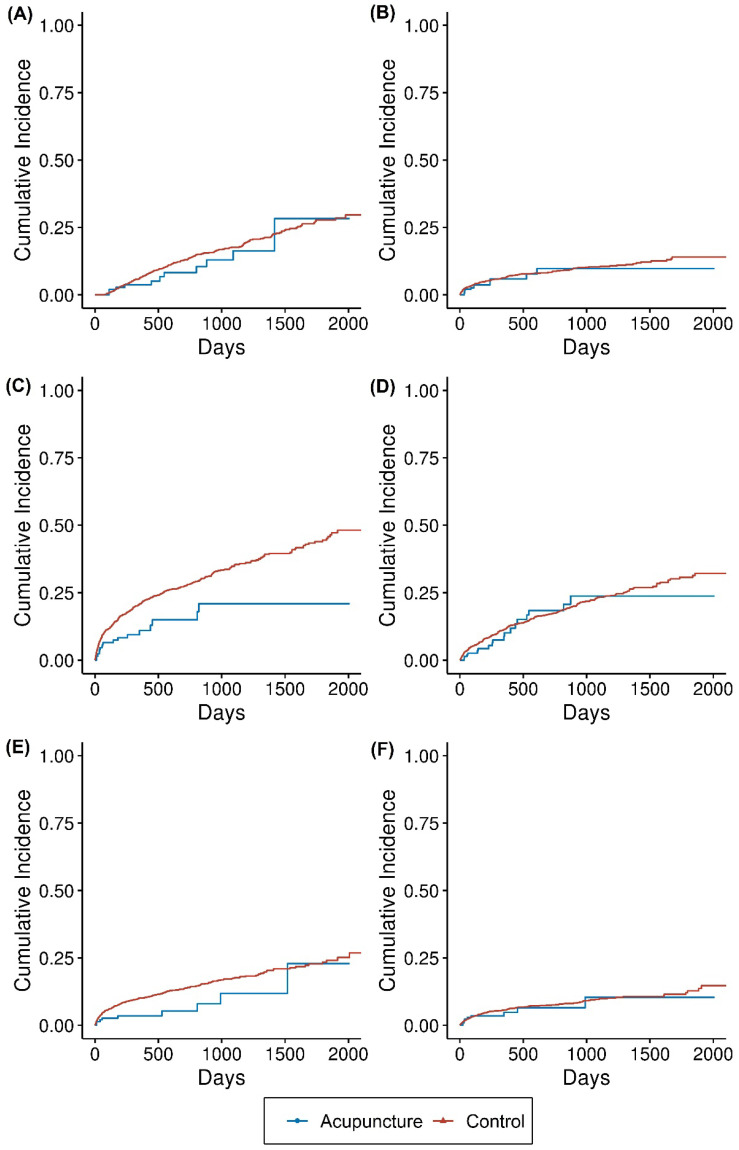
Extended Kaplan–Meier graph of the outcomes of the acupuncture and control groups. The cumulative incidence of each type of event is presented in the extended Kaplan–Meier graph. Events were (**A**) all-cause death, (**B**) readmission because of stroke relapse, (**C**) complications as a composite outcome, (**D**) pneumonia, (**E**) urinary tract infection, and (**F**) decubitus.

During the follow-up, the incidence of all-cause death was 13.6/100,000 person-days in the acupuncture group and 25.7/100,000 person-days in the control group. The incidence of readmission was 11.0/100,000 person-days in the acupuncture group and 13.1/100,000 person-days in the control group. The incidences of composite complications were 35.7/100,000 person-days in the acupuncture group and 55.1/100,000 person-days in the control group. There was a lower incidence of distinct complications in the acupuncture group than in the control group (Table 2). The cumulative incidence is presented in Figure 2.

Table 3 shows the crude and adjusted HR for all-cause death, readmission, and complications. The HR for all-cause death and complications were 0.32 (95% CI, 0.18–0.60) and 0.34 (95% CI, 0.21–0.53), respectively, indicating that acupuncture is associated with a lower risk of these outcomes. Among the complications, only the HR for UTI was significantly lower (HR: 0.24; 95% CI, 0.11–0.54); significant between-group differences were not detected for pneumonia, decubitus ulcer, or readmission (Table 3). These results were consistent with those of the matched cohort (Table S4). The results of the sensitivity analysis for the criteria used to define the acupuncture group (i.e., the number of acupuncture sessions) were mostly consistent with the results of the main analysis (Figure S2). The HR generally decreased as the number of sessions increased. The follow-up sensitivity analysis results were also consistent with the results of the main analysis (Figure S3).

## 4. Discussion

The present study investigated the utilization of acupuncture treatment and the association of acupuncture treatment with the prognosis for patients with subacute and chronic ischemic stroke using data from 2010 to 2015. We aimed to establish a model for acupuncture utilization based on its time-varying nature. Accordingly, we performed a time-dependent survival analysis. To the best of our knowledge, this is the first study of acupuncture using a time-dependent design. Because the reasons for the acupuncture session criteria used during previous retrospective cohort studies are unclear, we referenced several articles [20,21,22,23] when designing our sensitivity analysis and reported consistent results.

The HR of all-cause death was 0.32 (95% CI: 0.18–0.60), which was lower in the acupuncture group. Previous studies mostly investigated function rehabilitation or complication after acupuncture, and few studies investigated long-term mortality [11]. There has been one retrospective study of KM treatment [21], but it did not achieve definitive conclusions. Although the mortality rate was significantly lower in the matched cohort (HR: 0.44; 95% CI: 0.21–0.93), the results for moderate ischemic stroke patients became nonsignificant in the additionally adjusted model (HR: 0.55; 95% CI: 0.47–0.98). The present study used a time-dependent design to demonstrate the association between acupuncture utilization and mortality. Such findings were consistent in the adjusted matched cohorts. In the previous randomized controlled trial [7], the intention-to-treat analysis could not demonstrate the effect of acupuncture on mortality (OR: 0.75; 95% CI: 0.54–1.05); however, the per-protocol analysis showed a significant result (OR: 0.68; 95% CI: 0.47–0.98). Because the time-dependent design reflects patient compliance with acupuncture treatment over time, it is similar to a per-protocol analysis. Such findings suggested the need for measures to increase patient compliance in the real world.

We also analyzed the association of acupuncture treatment with readmission and complications. The HR of UTI was significantly lower (HR: 0.24; 95% CI: 0.11–0.54), which was consistent with the findings of a previous study in Taiwan (HR: 0.76; 95% CI: 0.73–0.80) [26]. Because UTI is associated with poorer neurological outcomes [8], this complication would have contributed to lower mortality rates. However, the results did not significantly correlate with readmission (HR: 0.53; 95% CI: 0.25–1.09), pneumonia (HR: 0.68; 95% CI: 0.44–1.04), and decubitus ulcer (HR: 0.48; 95% CI: 0.22–1.04). These findings were inconsistent with those of previous studies in Taiwan (recurrence, HR: 0.88; 95% CI: 0.84–0.91; pneumonia, HR: 0.86; 95% CI: 0.82–0.90) [22,27]; this could be due to differences in healthcare environment between different countries. For other reason, we applied more precise criteria to include subacute and chronic ischemic stroke patients and to reflect the healthcare environment in Korea. However, this led to lower number of participants than previous literature. Although our study showed lower estimates of hazard ratios, the confidence interval was excessively wider than previous studies. Additional studies with large samples are warranted.

Although the mechanism of this phenomenon has not been clearly identified, existing studies have proposed various hypotheses. From a physiologic perspective, acupuncture treatment can facilitate recovery from ischemic stroke through neurogenesis by enhancing the brain-derived neurotrophic factor and vascular endothelial growth factor signaling pathways [28,29]. Acupuncture has also been shown to effectively control circulation [30], including cerebral blood flow [31,32]. Its potential effectiveness in regulating nitric oxide synthesis [33] and angiotensin II and its receptor-mediated mechanism [34] may also affect poststroke outcomes. Such effects can enhance recovery from neurological impairment.

Clinical studies have shown that acupuncture can increase the functional ability of neurons and improve neurological impairments, such as global neurological deficiency and spasticity [11,35,36]. Moreover, acupuncture can effectively manage chronic pain [37] experienced by chronic stroke patients [11] and prevent critical complications such as dysphagia [38] experienced by stroke patients. These effects can contribute to the long-term outcomes of acupuncture.

This study had several limitations. For example, there may have been uncontrolled confounders. Because of the limitations of using insurance claims data, information about mobility and the ability to perform activities of daily living was limited. Future prospective studies should investigate these issues. Moreover, time-varying confounders may have existed. We selected confounders based on the baseline information. However, as follow-up time goes on, it is possible that only people who survived are allocated to the acupuncture group. We performed a sensitivity analysis based on the follow-up time and the results were consistent. To completely control the differences, time-varying confounders must be adjusted; however, selecting appropriate time-varying confounders was considered difficult because of the nature of the data. Finally, it was impossible to perform subgroup analyses and investigate various outcomes because of the relatively small number of events and patients in the acupuncture group.

The analysis performed during the present study included all acupuncture treatment modalities used in the real world. Such an analysis has the advantage of being pragmatic. However, it is necessary to establish a standardized acupuncture protocol for subsequent prospective studies using detailed information about the techniques and dosage of acupuncture. Although we compiled a list of the main diagnoses for which the acupuncture treatment was applied, the specific composition (e.g., dosage) of acupuncture treatment could not be determined.

## 5. Conclusions

Because of the time-varying characteristics of acupuncture, it might be associated with lower mortality rates and the prevention of complications after ischemic stroke. This study demonstrated that adherence to acupuncture treatment must be increased. Further prospective studies are needed to establish our findings.

## Figures and Tables

**Table 1:** Baseline characteristics of patients.

Baseline Characteristics	Control Group	Acupuncture Group
	(*n* = 2104; 91.5%)	(*n* = 195; 8.5%)
Sex		
Female	892 (42.4)	82 (42.1)
Male	1212 (57.6)	113 (57.9)
Age		
Age in years	68.5 ± 11.8	69.0 ± 11.1
40–50	137 (6.5)	9 (4.6)
50–60	388 (18.4)	35 (17.9)
60–70	500 (23.8)	41 (21.0)
70–80	661 (31.4)	79 (40.5)
80 and above	418 (19.9)	31 (15.9)
Income		
High	831 (39.5)	81 (41.5)
Low	608 (28.9)	50 (25.6)
Mid	665 (31.6)	64 (32.8)
Residence		
Metropolitan	842 (40.0)	84 (43.1)
Rural	345 (16.4)	25 (12.8)
Urban	917 (43.6)	86 (44.1)
Insurance		
Beneficiary	186 (8.8)	16 (8.2)
Nonworker	1270 (60.4)	110 (56.4)
Worker	648 (30.8)	69 (35.4)
Disability	342 (16.3)	31 (15.9)
Comorbidity ^†^		
CCI	4.3 ± 2.4	4.7 ± 2.1
Diabetes	1124 (53.4)	109 (55.9)
Hypertension	1665 (79.1)	167 (85.6)
Hyperlipidemia	1524 (72.4)	143 (73.3)
Renal failure	105 (5.0)	10 (5.1)
Heart failure	202 (9.6)	24 (12.3)
Cancer	644 (30.6)	63 (32.3)
Mental disorder	1117 (53.1)	124 (63.6)
Cardiac arrhythmia	130 (6.2)	19 (9.7)
Osteoarthritis	709 (33.7)	68 (34.9)
Rheumatoid arthritis	99 (4.7)	12 (6.2)
Lumbar disc herniation	225 (10.7)	18 (9.2)
Pneumonia	290 (13.8)	34 (17.4)
Urinary tract infection	186 (8.8)	22 (11.3)
Intervention ^‡^		
Admission in tertiary hospitals	720 (34.2)	70 (35.9)
Admission day > 7	1328 (63.1)	149 (76.4)
Nasogastric intubation	327 (15.5)	54 (27.7)
Urinary catheterization	579 (27.5)	86 (44.1)
ICU stay	233 (11.1)	40 (20.5)
Readmission after onset	163 (7.7)	30 (15.4)

Data from one month since the onset of stroke (cohort entry) were used and presented according to the treatment group allocation status within 3 months from cohort entry. Subsequent treatment allocation was varied in a time-dependent way. Continuous variables are expressed as mean ± standard deviation and categorical variables are expressed as *n* (%). ^†^ Information from 1 year prior to cohort entry was used. ^‡^ Intervention was defined according to the procedure the patients underwent at the time of admission for the first stroke onset; however, readmission was defined as hospitalization for at least 1 day with a primary diagnosis of ischemic stroke during the time period between the onset of the first stroke and cohort entry. CCI: Charlson comorbidity index; ICU: intensive care unit.

**Table 2:** Number of cases and incidence of outcomes in the acupuncture and control groups.

Outcomes	Cases (*n*)			Incidence (100,000 Person-Days)		
	Total	Acupuncture	Control	Total	Acupuncture	Control
All-cause death	389	11	378	25.1	13.6	25.7
Readmission	190	8	182	13	11	13.1
Composite of complications	664	20	644	54.2	35.7	55.1
Pneumonia	422	23	399	30.9	33.8	30.7
Urinary tract infection	325	8	317	23.4	11.7	24
Decubitus ulcer	213	9	204	14.5	12.4	14.6
Gastrointestinal hemorrhage	48	1	47	3.1	1.2	3.2
Femur fracture	48	1	47	3.1	1.3	3.2

The number of cases and the incidence according to the group are presented. Cases are presented as numbers, and the incidence rate is presented per 100,000 person-days.

**Table 3:** Comparison of hazard ratios for outcomes of the acupuncture and control groups.

Outcomes	Crude HR (95% CI)	Multivariate-Adjusted HR
All-cause death	0.48 (0.26–0.88)	0.32 (0.18–0.60)
Readmission	0.79 (0.39–1.62)	0.53 (0.25–1.09)
Composite complications	0.62 (0.40–0.97)	0.34 (0.21–0.53)
Pneumonia	1.09 (0.71–1.66)	0.68 (0.44–1.04)
Urinary tract infection	0.46 (0.23–0.94)	0.24 (0.11–0.54)
Decubitus ulcer	0.78 (0.40–1.53)	0.48 (0.22–1.04)

The reference group in the model is the control group. CI: confidence interval; HR: hazard ratio.

## Data Availability

The NHIS-NSC 2.0 is provided by the NHIS in Korea (https://nhiss.nhis.or.kr/bd/ay/bdaya001iv.do, accessed on 20 February 2022). To protect privacy, access to the data is available only to certified researchers in South Korea.

## Supplementary Materials

The following supporting information can be downloaded at: https://www.mdpi.com/article/10.3390/healthcare10050756/s1, Figure S1: Design of the present study; Figure S2: Sensitivity analysis with various criteria; Figure S3: Sensitivity analysis with various observation periods; Table S1: Code for definition of acupuncture, complications and other medical intervention; Table S2: Example of the study design; Table S3: The list of diagnoses for which acupuncture treatment was performed; Table S4: Sensitivity analysis with a matched cohort.
