# Supplementary material for: A Time-Dependent Analysis of Association between Acupuncture Utilization and the Prognosis of Ischemic Stroke
Source: Healthcare (Basel). 2022 Apr 19;10(5):756. doi: 10.3390/healthcare10050756 (PMC9141209; doi:10.3390/healthcare10050756)
Supplement: Supplementary file 1 [file healthcare-10-00756-s001.zip › healthcare-1626673-supplementary.pdf]

Table S1. Code for definition of acupuncture, complications and other medical intervention

Category	Definition	Code type	Code
Acupuncture	Single body part	Reimbursement code	40011
	Multiple body parts	Reimbursement code	40012
	Intra-orbital cavity	Reimbursement code	40030
	Intranasal sinus	Reimbursement code	40040
	Intra-peritoneal cavity	Reimbursement code	40050
	Intra-articular	Reimbursement code	40060
	Interspace of spinous process or transverse process	Reimbursement code	40070
	Penetration	Reimbursement code	40080
	Electro-acupuncture	Reimbursement code	40091
Complications	Pneumonia	KCD - 7	J12 – J18, J69, J841, J849
	Urinary tract infection	KCD - 7	N390
	Decubitus ulcer	KCD - 7	L89
	Gastrointestinal bleeding	KCD - 7	K922
	Femur fracture	KCD - 7	S72
Other medical interventions	Nasogastric intubation	Reimbursement code	Q2621, Q2622
	Urinary catheterisation	Reimbursement code	R3381, M0050, M0060, M0065
	ICU stay	Reimbursement code	AJ001, AJ002, AJ003, AJ006, AJ007, AJ110, AJ200, AJ300, AJ500, 19001, 19002, 19003, 19006, 19007, 19200, 19300, 19400, 19500

KCD - 7: Korean Classification of Diseases, Seventh Revision

Table S2. Example of the study design

ID	Start	Stop	Time window	Group assignment	Cumulative number of acupuncture sessions during each time window	ID	Start	Stop	Time window	Group assignment	Cumulative number of acupuncture sessions during each time window
A	0	1	1	Control	0	C	0	1	1	Control	0
A	1	2	1	Control	0	C	1	2	1	Control	0
A	2	3	1	Acupuncture	1	C	2	3	1	Control	0
A	3	4	1	Acupuncture	2	C	3	4	1	Control	0
A	...	...	...	...	...	C	...	...	...	...	...
A	88	89	1	Acupuncture	12	C	88	89	1	Control	0
A	89	90	1	Acupuncture	13	C	89	90	1	Control	0
A	90	91	2	Control	0	C	90	91	2	Control	0
A	91	92	2	Control	0	C	91	92	2	Control	0
A	92	93	2	Acupuncture	1	C	92	93	2	Acupuncture	1
A	93	94	2	Acupuncture	2	C	93	94	2	Acupuncture	2
A	...	...	...	...	...	C	...	...	...	...	...
A	178	179	2	Acupuncture	12	C	178	179	2	Acupuncture	12
A	179	180	2	Acupuncture	13	C	179	180	2	Acupuncture	13
B	0	1	1	Control	0	D	0	1	1	Control	0
B	1	2	1	Control	0	D	1	2	1	Control	0
B	2	3	1	Acupuncture	1	D	2	3	1	Acupuncture	1
B	3	4	1	Acupuncture	2	D	3	4	1	Acupuncture	2
B	...	...		...	...	D	...	...		...	...
B	87	88	1	Acupuncture	11	D	87	88	1	Acupuncture	11
B	88	89	1	Acupuncture	12	D	88	89	1	Acupuncture	12
B	89	90	1	Acupuncture	13	D	89	90	1	Acupuncture	13
B	90	91	2	Control	0	D	90	91	2	Control	0
B	91	92	2	Control	0	D	91	92	2	Control	0
B	92	93	2	Control	0	D	92	93	2	Control	0
B	93	94	2	Control	0	D			Control	2	1
B	...	...		...	...	D	Censored		...	...	...
B	178	179	2	Control	0	D			Control	2	10
B	179	180	2	Control	0	D			Control	2	11

Observation began at 1 month after stroke onset; hence, the Start column represents time zero. The time window was set to 3 months. "A" received acupuncture 2 days after time zero and received at least 13 sessions of acupuncture during the first time window. Accordingly, "A" was assigned to the acupuncture group starting from the time of the first acupuncture treatment. "A" satisfied the acupuncture criteria during the second time window; therefore, "A" was assigned to the acupuncture group. For robustness, the period prior to assignment included the control group.

"B" satisfied the criteria during the first time window but did not receive any acupuncture treatment during the second time window; therefore, "B" was assigned to the control group. "C" did not receive any acupuncture treatment during the first time window and was assigned to the control group; however, "C" was subsequently assigned to the acupuncture group during the second time window.

"D" received acupuncture treatment during the second time window but did not satisfy the criteria. This was considered a protocol violation; therefore, data after the first acupuncture treatment were censored.

Table S3. The list of diagnoses for which acupuncture treatment was performed

Patients			Sessions		
Diagnosis	KCD-7	n (%)	Diagnosis	KCD-7	n (%)
Sequelae of cerebral infarction	I693	86 (24.8)	Sequelae of wind stroke disorder	U234	2,312 (20.8)
Sequelae of wind stroke disorder	U234	78 (22.5)	Sequelae of cerebral infarction	I693	1,232 (11.1)
Low back pain	M545	64 (18.4)	Low back pain	M545	940 (8.5)
Cerebral infarction, unspecified	I639	58 (16.7)	Cerebral infarction, unspecified	I639	482 (4.3)
Hemiplegia, unspecified	G819	32 (9.2)	Myalgia	M791	382 (3.4)
Myalgia	M791	30 (8.6)	Flaccid hemiplegia	G810	314 (2.8)
Gonarthrosis, unspecified	M179	21 (6.1)	Muscle strain	M626	279 (2.5)
Muscle strain	M626	20 (5.8)	Gonarthrosis, unspecified	M179	257 (2.3)
Limb pain	M796	20 (5.8)	Hemiplegia, unspecified	G819	237 (2.1)
Joint pain	M255	14 (4)	Sequelae of other and unspecified cerebrovascular diseases	I698	228 (2.1)

A total of 347 patients were assigned to the acupuncture group during the total observational period: 195 were assigned to the acupuncture group at baseline and 152 more were subsequently assigned to the acupuncture group. A total of 11,106 acupuncture sessions were performed. "Wind stroke" is the medical term for stroke in the field of Korean traditional medicine.

Table S4. Sensitivity analysis with a matched cohort

	Multivariate-adjusted HR after propensity score matching (95% CI)
**All-cause death**	0.26 (0.12–0.56)
**Readmission**	0.53 (0.20–1.41)
**Composite complications**	0.22 (0.12–0.43)
Pneumonia	0.62 (0.33–1.17)
Urinary tract infection	0.25 (0.07–0.87)
Decubitus ulcer	0.40 (0.13–1.27)

The acupuncture and control groups were propensity score-matched using a ratio of 1:2. The reference group in the model is the control group.

CI, confidence interval; HR, hazard ratio.

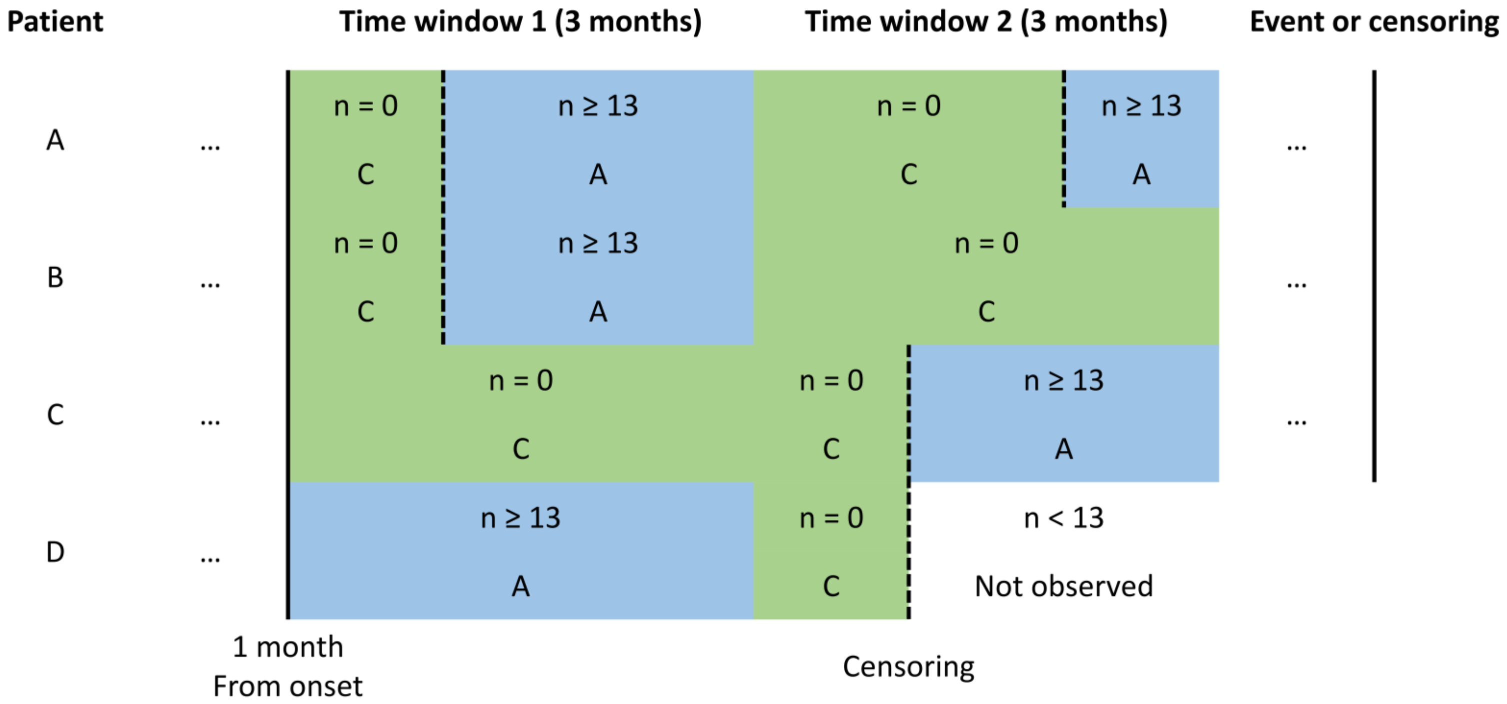

Figure S1. Design of the present study

A time-dependent design was adapted in this study. The number of acupuncture treatment sessions that patients received every 3 months was evaluated. Patients were assigned to the acupuncture group according to the number of acupuncture sessions they received. The time before the first treatment session in each time window was defined as the control period. The control group was defined as patients who did not receive acupuncture during each time window. If the patients had received any acupuncture, then their data were censored. n: number of treatment sessions; A: acupuncture group; C: control group. The first treatment session in the time window is represented by a dotted line.

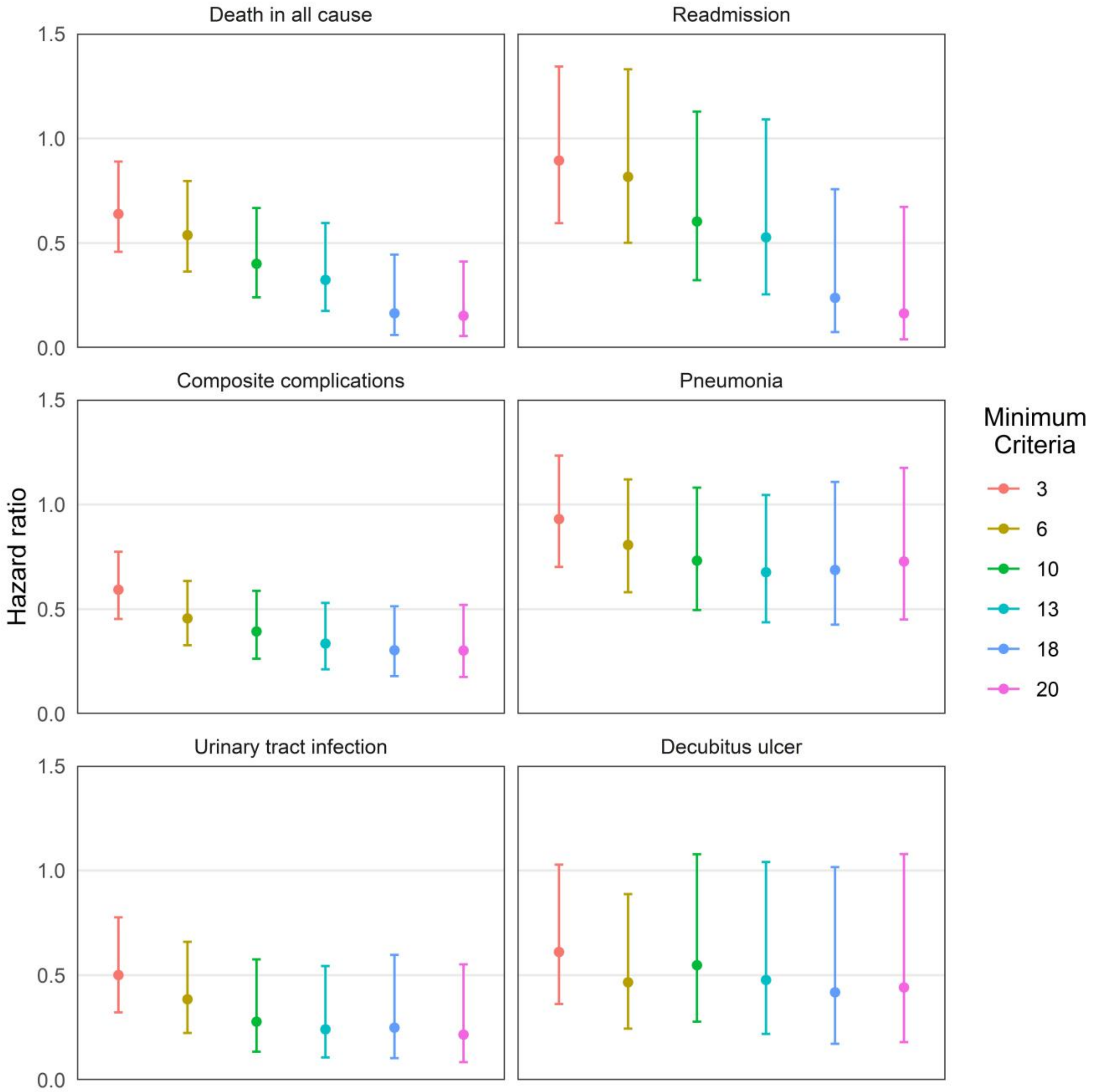

Figure S2. Sensitivity analysis with various criteria

A sensitivity analysis was performed by varying the minimum criteria (i.e., number of acupuncture sessions received during each time window) used to define the acupuncture group. The criteria were as follows: three sessions, the criteria used by Lee et al.[1]; six sessions, the median of the cumulative number of acupuncture treatments received during each time window among patients prescribed acupuncture, as per Shih et al.[2]; 10 sessions, half the number of sessions used by Wayne et al.[3]; 13 sessions, the criteria used in the main analysis assuming patients were prescribed acupuncture more than once per week during the 3-month period; 18 sessions, the 75th percentile of the cumulative number of prescribed acupuncture treatments during each time interval among patients prescribed acupuncture; and 20 sessions, the criteria used by Wayne et al.[3].

1. Lee Y-S, Kwon S, Chae Y, et al. A retrospective cohort study on the outcomes of ischemic stroke patients with adjuvant Korean Medicine treatment. Scientific reports 2018; 8: 1-8.

2. Shih C-C, Liao C-C, Sun M-F, et al. A retrospective cohort study comparing stroke recurrence rate in ischemic stroke patients with and without acupuncture treatment. Medicine 2015; 94.

3. Wayne PM, Krebs DE, Macklin EA, et al. Acupuncture for upper-extremity rehabilitation in chronic stroke: a randomized sham-controlled study. Archives of physical medicine and rehabilitation 2005; 86: 2248-2255.

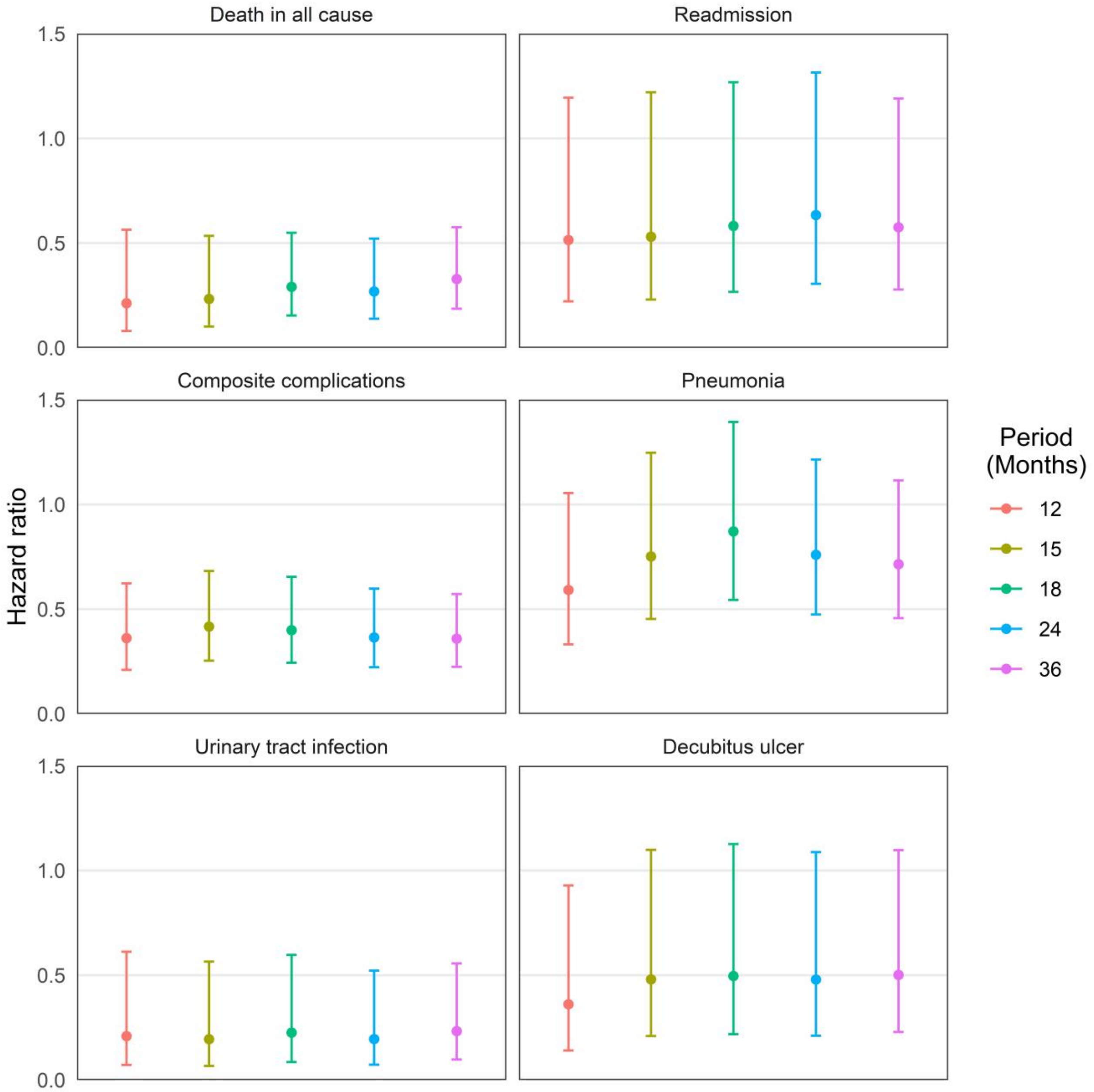

Figure S3. Sensitivity analysis with various observation periods

To exclude patients who would receive acupuncture treatment only if the severity increased over time, the analysis was performed in the same manner as the main analysis, but the observation period was varied.
